# Supplementary material for: Nanopore sequencing reveals that DNA replication compartmentalisation dictates genome stability and instability in Trypanosoma brucei
Source: Nat Commun. 2025 Jan 16;16:751. doi: 10.1038/s41467-025-56087-3 (PMC11739655; doi:10.1038/s41467-025-56087-3)
Supplement: Supplementary file 1 — Supplementary information [file 41467_2025_56087_MOESM1_ESM.pdf]

Supplementary Information

Nanopore sequencing reveals that DNA replication compartmentalisation dictates genome stability and instability in *Trypanosoma brucei*

Marija Krasiļņikova[1], Catarina A. Marques[1*], Emma M. Briggs[1,2,3], Craig Lapsley[1], Graham Hamilton[4], Dario Beraldi[1], Kathryn Crouch[1] and Richard McCulloch[1*]

1. University of Glasgow Centre for Parasitology, The Wellcome Centre for Integrative Parasitology, University of Glasgow, School of Infection and Immunity, Sir Graeme Davies Building, 120 University Place, Glasgow, G12 8TA, United Kingdom.

2. University of Edinburgh, Institute for Immunology and Infection Research, School of Biological Sciences, Edinburgh, United Kingdom

3. Biosciences Institute, Cookson Building, Newcastle University, Framlington Place, Newcastle upon Tyne, NE2 4HH, United Kingdom.

4. MVLS Research Facilities, University of Glasgow, Wolfson Wohl Cancer Research Centre, Garscube Estate, Switchback Rd, Bearsden, G61 1QH, United Kingdom

*Correspondence to:

Richard.mcculloch@glasgow.ac.uk; Catarina.DeAlmeidaMarques@glasgow.ac.uk

Sequencing statistics

Total number of reads sequenced	319075
Total bases sequenced	3 558 525 709
Mean read length (bp)	11 152.6
Median read length (bp)	4307
Longest reads (bp)	345 688
	277 178
	252 902
	241 633
	240 202

>50 kb assembly read coverage

Min. coverage	0
Max. coverage	72
25th percentile (Q1)	11
Median (Q2)	17
75 percentile (Q3)	25
Interquartile range	14
Mean coverage	18
Standard deviation	9.6
Percentage of assembly covered by at least 1 >50kb read	98.47%
Percentage of assembly covered by at least 10 >50 kb reads	79.76%

Supplementary Table 1. Nanopore sequencing of the *T. brucei* Lister 427 genome.

Genome statistics	NANOPORE 427	427 2018	427
# contigs	166	317	32
# contigs (>= 10000 bp)	104	303	32
# contigs (>= 25000 bp)	100	183	31
# contigs (>= 50000 bp)	97	72	24
largest contig	5080222	4633729	4977113
total length	55332974	50081021	26754408
total length (>= 25000 bp)	55128177	47671040	26743874
total length (>= 50000 bp)	54988747	43945746	26465291
N50	2194184	1412180	2482252
N75	538565	539199	1619978
L50	9	11	4
L75	22	25	7
GC (%)	43.85	43.71	46.71
# VSG genes	3511	3524	387
BUSCO score	C: 139 [S: 128, D: 11], F: 23, M: 93, n: 255	C: 141 [S: 138, D: 3], F: 22, M: 92, n: 255	C: 140 [S: 137, D: 3], F: 22, M: 93, n: 255

Supplementary Table 2. Comparing genome assembly of the Lister 427 *T. brucei* genome by Nanopore and PacBio/HiC analysis, as well as the original *T. brucei* 427 reference genome.

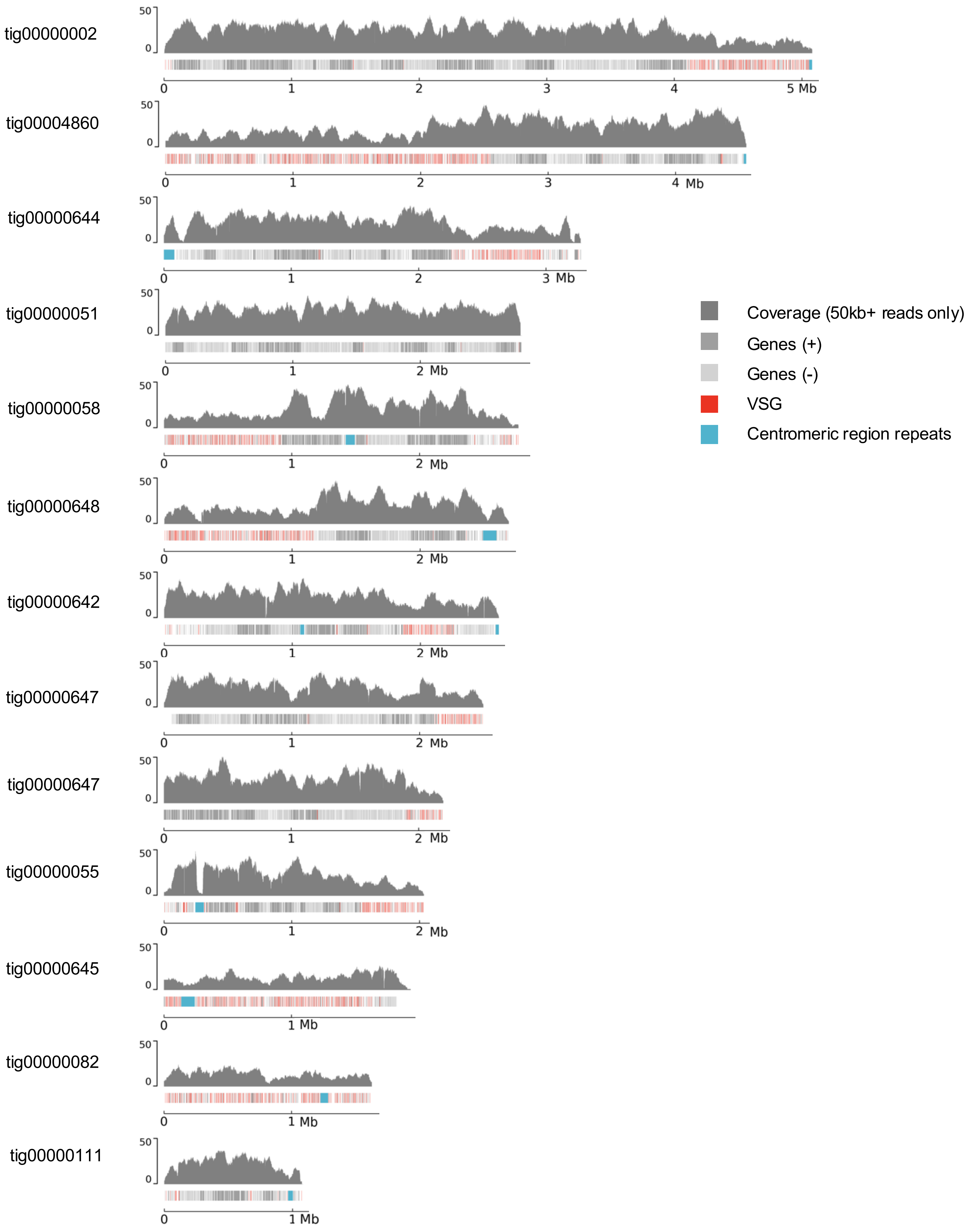

Supplementary Figure 1. Nanopore contigs greater than 1 Mb in size. 13 Nanopore contigs (tigs) are shown in which core genes are shown organised as directional gene clusters (dark grey and light depicting the transcribed strand), centromeres are shown in blue, and *VSG*s within the subtelomeres in red. Mapped read depth of Nanopore reads >50 kbp in length are shown for each contig.

Chr	Contigs within the reference					Contigs bridged in the new assembly
	**5A**	**5B**	**3A**	**3B**	**core**	
**1**	✓	✓	✓	✓	✓	3A & core<br>3B & core<br>5B & core<br>5A & core
**2**	✓				✓	
**3**	✓	✓	✓		✓	5A & core & 3A<br>5B & core
**4**	✓	✓	✓	✓	✓	5A & 5B & core & 3A<br>3B & core
**5**			✓	✓	✓	3A & core (& BES5)<br>3B & core
**6**			✓	✓	✓	3B & core<br>3A & core
**7**	✓				✓	5A & core
**8**	✓	✓	✓	✓	✓	5B & core<br>3A & core<br>5A & core
**9**	✓	✓	✓	✓	✓	3B & core<br>3A & core<br>5A & core
**10**	✓	✓	✓	✓	✓	3B & core<br>3A & core<br>5B & core
**11**	✓	✓	✓	✓	✓	3B & core

Supplementary Table 3. Summary of Nanopore assembly of the 11 *T. brucei* megabase chromosomes.

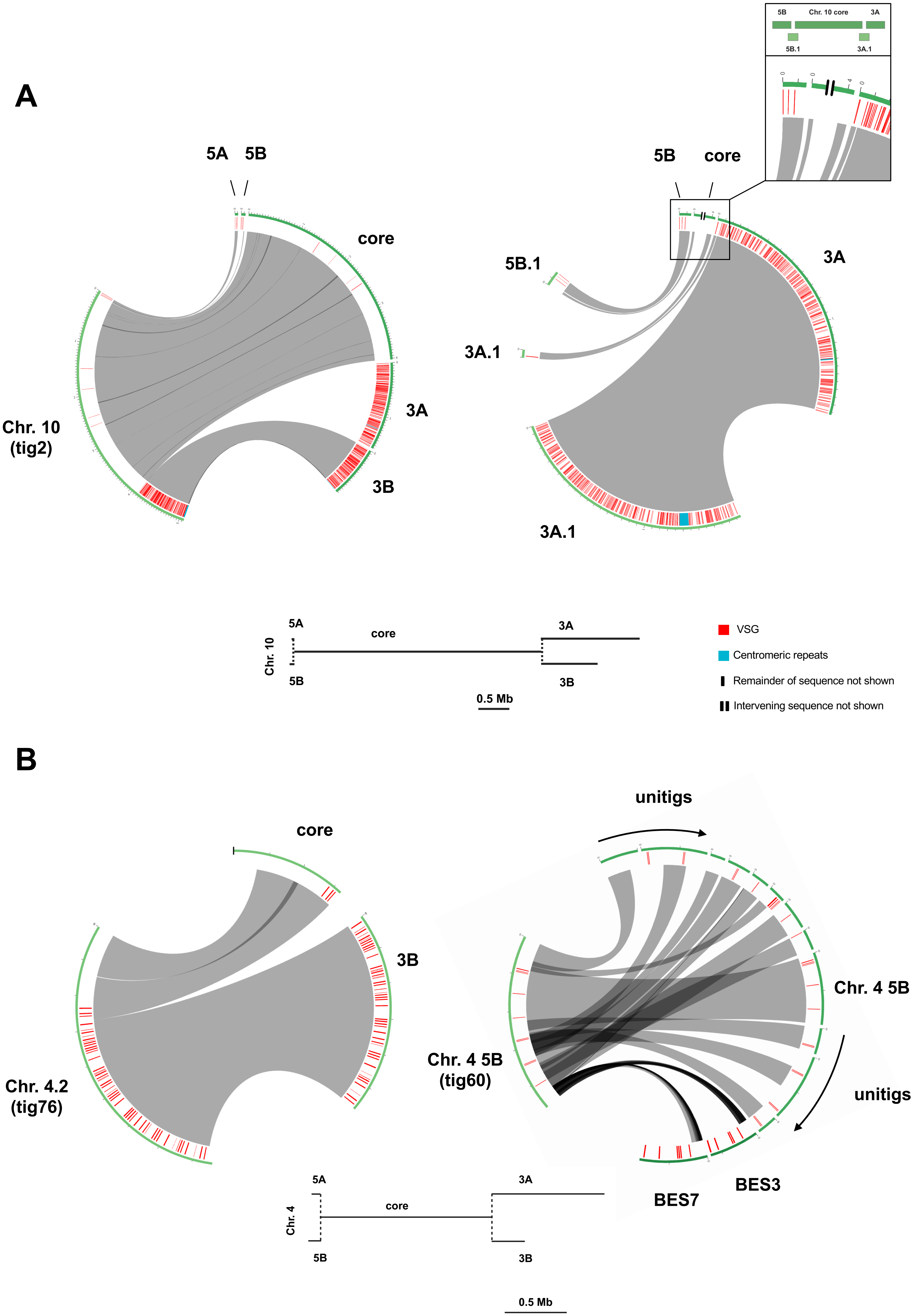
A
5A
5B
core
3A
3B
Chr. 10
(tig2)
5B.1
3A.1
Chr. 10 core
0.5 Mb
VSG
Centromeric repeats
Remainder of sequence not shown
Intervening sequence not shown
B
core
3B
Chr. 4.2
(tig76)
unitigs
Chr. 4 5B
Chr. 4 5B
(tig60)
BES3
BES7
5A
3A
Chr. 4
5B
3B
0.5 Mb

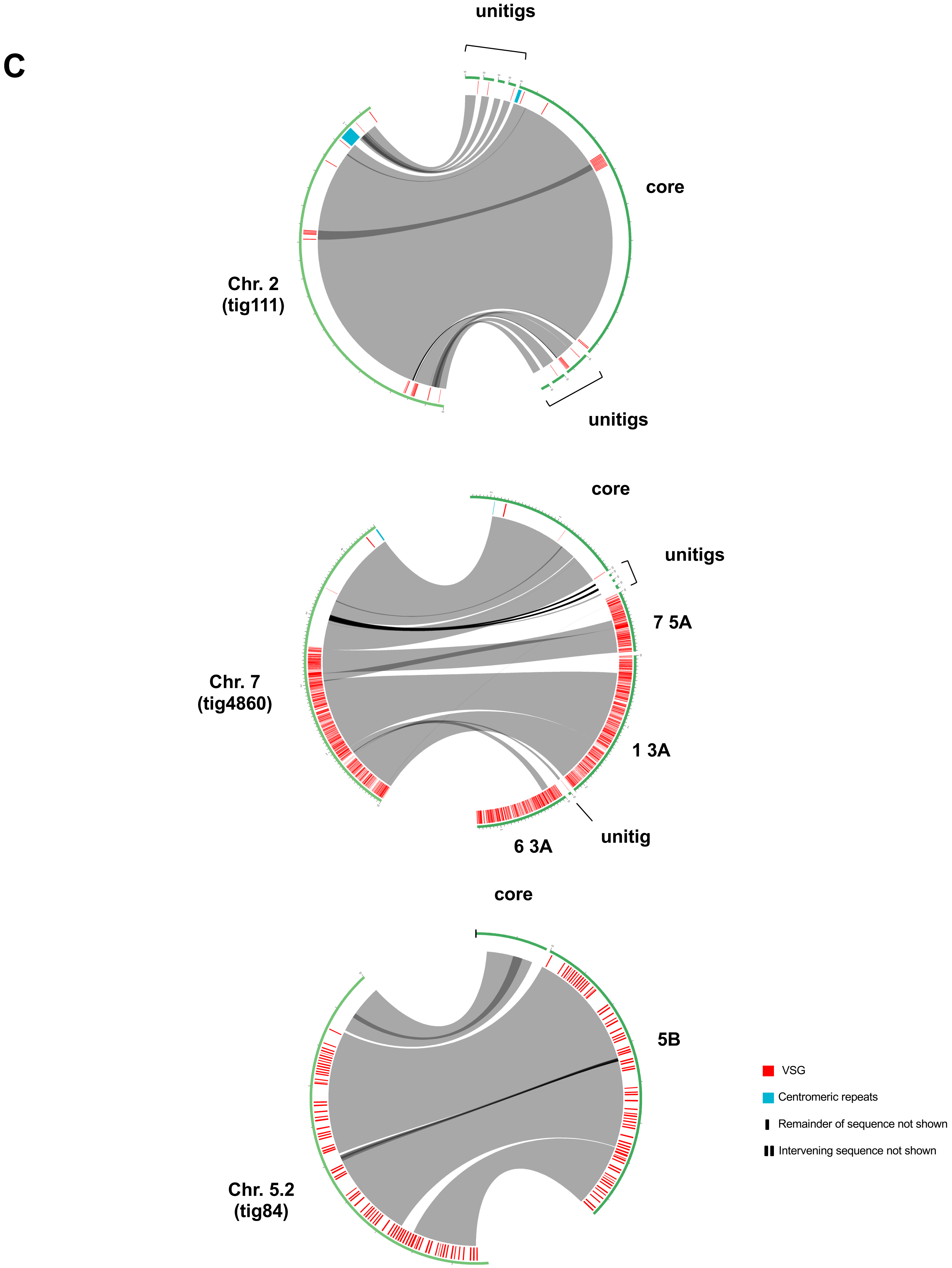

Supplementary Figure 2. Connecting the cores, subtelomeres and VSG expression sites of the *T. brucei* megabase chromosomes through Nanopore long-read sequencing. Circos plots highlighting synteny between

the Muller genome assembly and Nanopore contigs (tigs) of chromosome 10 (A), chromosome 4 (B), and three further chromosomes (C). In all panels grey ribbons represent overlaps, black ribbons represent multiple overlaps within a region, red denotes *VSG* genes, and blue denotes a centromere; in each case, the transcribed core, transcriptionally silent subtelomeres (numbered 3A, 3B, 5A, 5B), and bloodstream VSG expression sites (BESs) in the Muller genome are labelled, with the organisation of these compartments diagrammed (adapted from [9]). Elements of the figure were created using BioRender (McCulloch, R. (2025) https://biorender.com/j59e254, https://BioRender.com/h64v762).

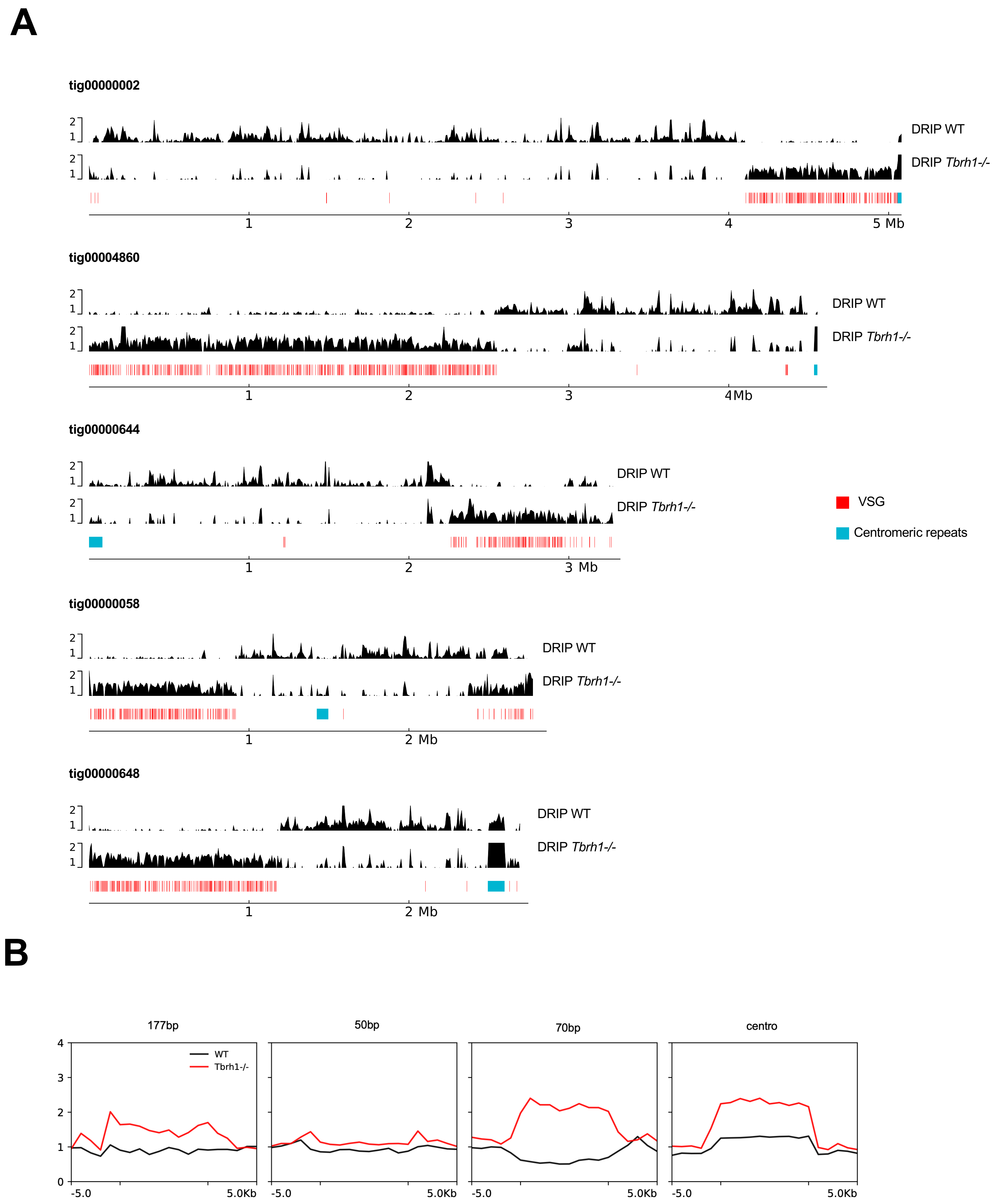

Supplementary Figure 3. R-loop mapping across the megabase chromosomes and DNA repeat regions of the *T. brucei* genome. A. Mapping of R-loops (DNA-RNA hybrid immunoprecipitation and sequencing, DRIP; data shown as IP/input) in wild type (WT) bloodstream from cells and RNase H1 null (-/-) mutants is shown in five Nanopore contigs (tigs) that span the core and subtelomere compartments. **B.** Metaplots of DRIP-seq signal in WT and RNase H1-/- cells across all Nanopore assembled regions containing 177 bp, 50 bp, 70 bp and centromeric (centro) repeats.

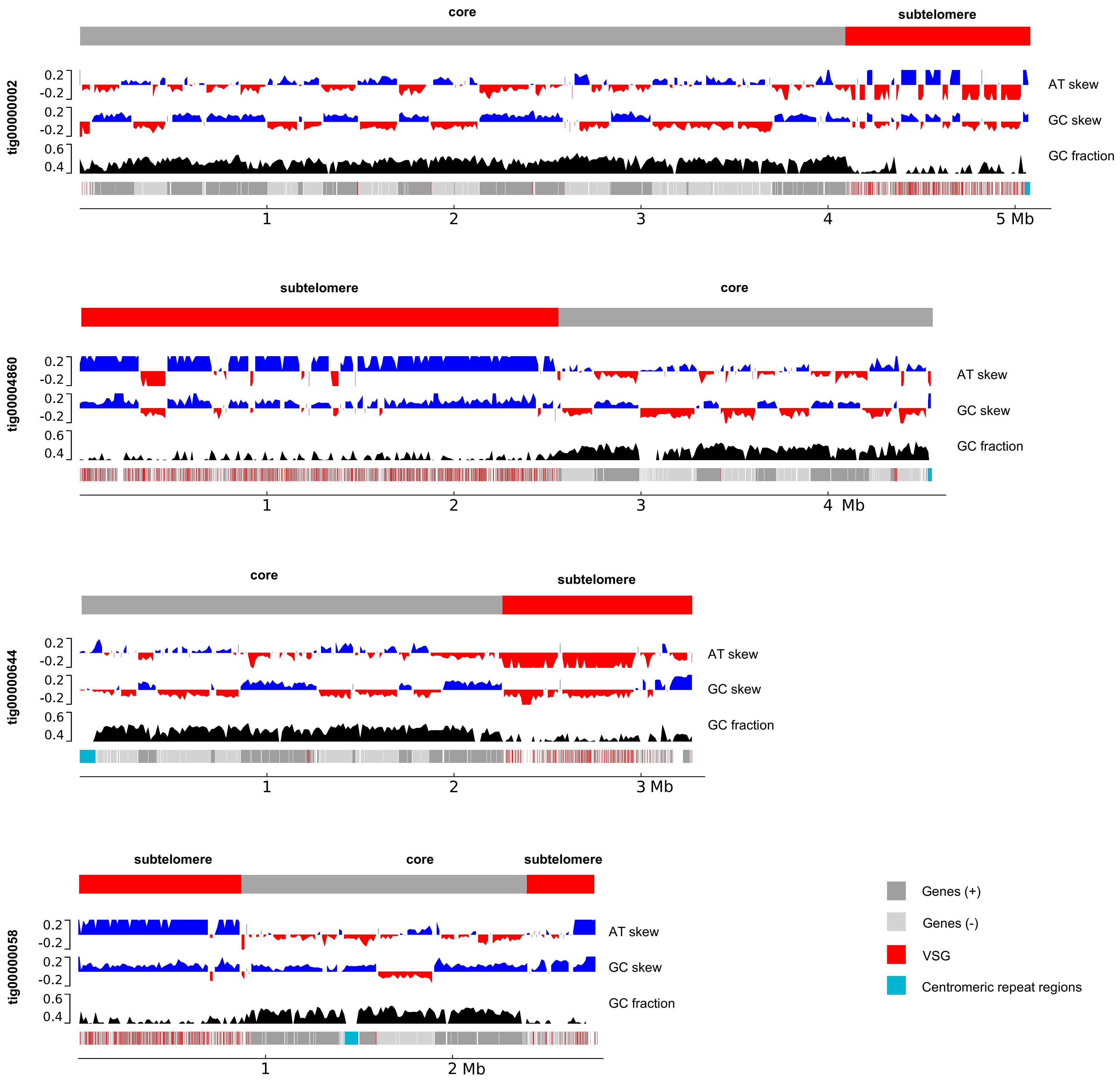

Supplementary Figure 4. Distinct base composition of the core and subtelomeres of the *T. brucei* megabase chromosomes. GC skew, AT skew and GC content is shown for four Nanopore contigs (tigs) that span the core (grey) and subtelomeric (red) compartments of *T. brucei* megabase chromosomes. Base skew is shown relative to transcribed strands of directional gene clusters (blue, forward strand; red, reverse strand).

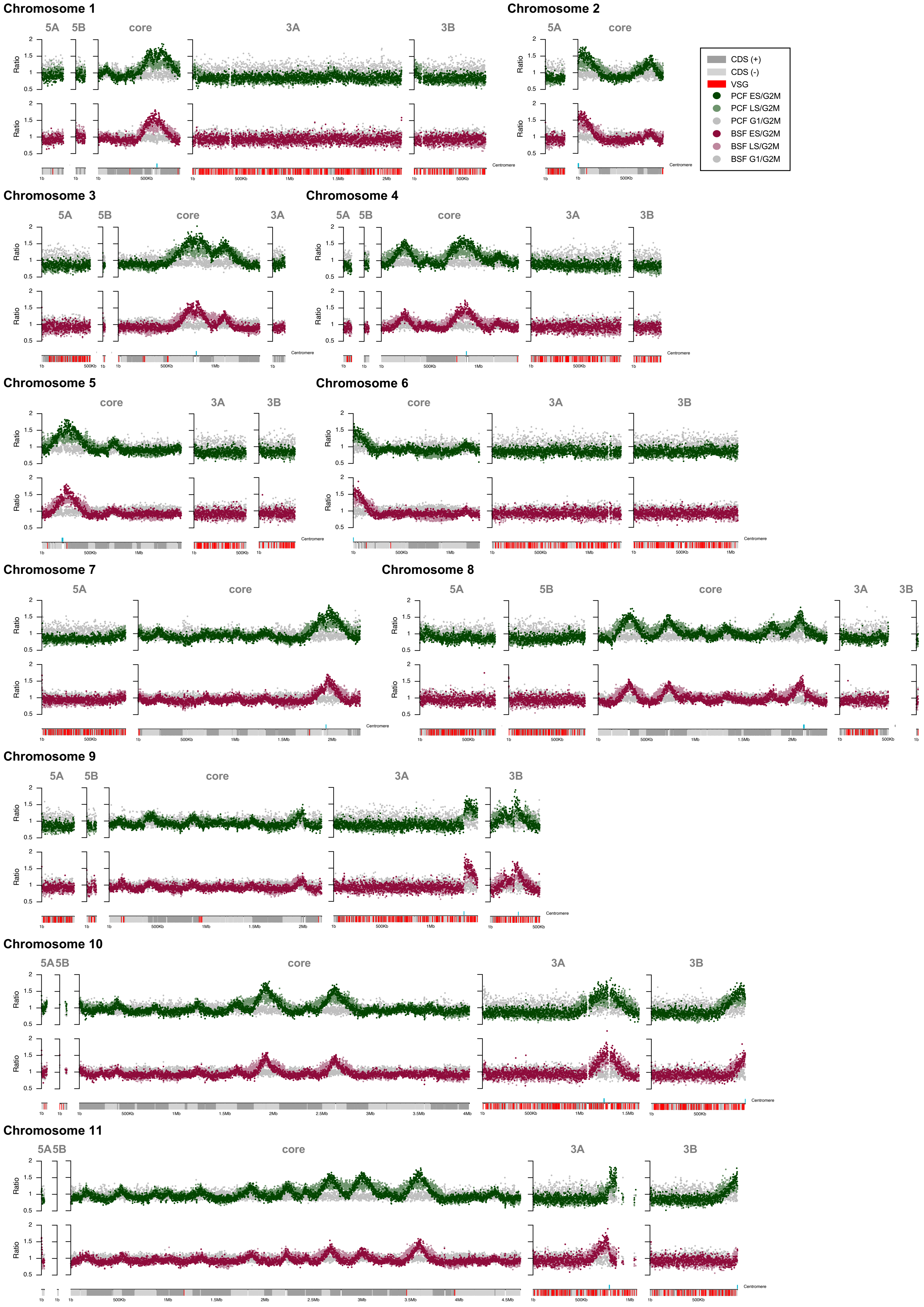

Supplementary Figure 5. Compartmentalisation of DNA replication between the cores and subtelomeres of the *T. brucei* megabase chromosomes. The distribution of DNA replication origins across the core and associated subtelomeres of the 11 megabase chromosomes, assessed by MFA-seq , is shown using the Muller genome

(tritrypDB, release v46) as a reference. Directional gene clusters in each chromosome core (dark grey and light depicting the transcribed strand), centromeres (blue), and *VSG*s (red) within the subtelomeres are indicated. The graphs above each chromosome show the read depth ratio of Illumina sequence derived from DNA of the following cells: early S phase/G2-M phase cells (dark red, bloodstream cells, BSF; dark green, procyclic form, PCF), late S/G2M (light red, BSF; light green, PCF), and G1/G2M (grey for both life cycle stages); each point represents the median S/G2M or G1/G2 ratio (y-axis) in 1 kb bins across the contig. All graphs are scaled according to chromosome compartment size. Source data are provided as a Source Data file.

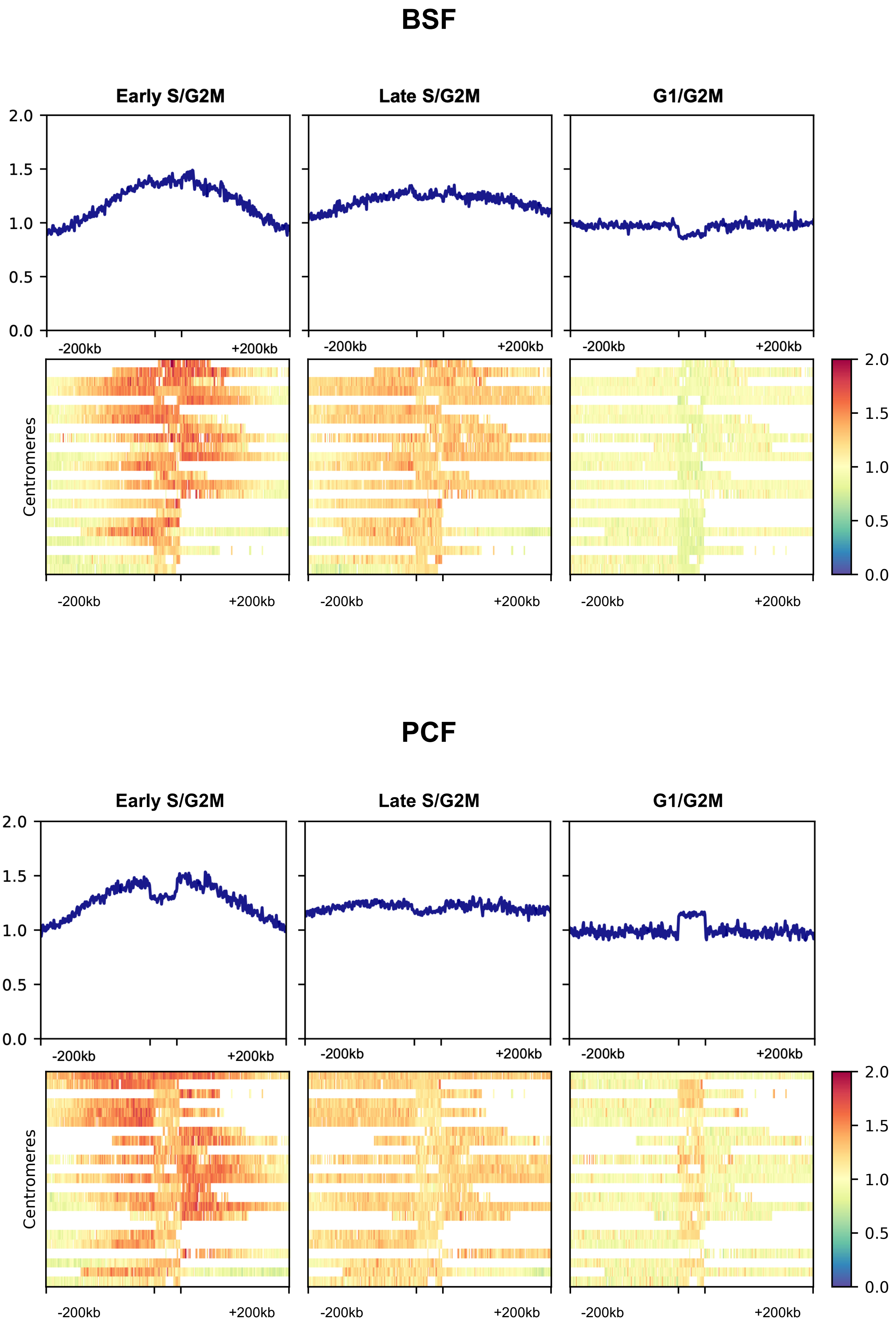

Supplementary Figure 6. DNA replication initiation at all assembled *T. brucei* centromeres. Metaplots and heatmaps of MFA-seq signal across centromere candidates and flanking sequence up to 200 kb upstream and downstream. BSF, bloodstream- form cells; PCF, procyclic form cells. Signal is the ratio of read depth in early S-phase, late S-phase or G1-phase cells relative to G2-M cells. In the heatmaps where there is not enough sequence data upstream or downstream, the region is coloured white.

NO	CONTIG	CHR	SIZE (KB)	PUBLISHED SIZE (KB)	FULL-LENGTH	AT CONTENT (%)	PUBLISHED AT CONTENT (%)
**1**	tig00000654	1	20.4	20 & 65	No	70	66
**2**	tig00000069	1	21.3		No	69	
**3**	tig00000111	2	37.2	30 & 55	Yes	73	66
**4**	tig00000126	2	36.6		Yes	72	
**5**	tig00000642a	3	30.2	75 & 80	Yes	50	49
**6**	tig00000058	4	70.6	70	Yes	62	61
**7**	tig00000055	5	66.5	50 & 75	Yes	62	62
**8**	tig00000648	6	103.7	55	Yes	70	71
**9**	tig00000127	7	17.0	100 & 120	No	73	75
**10**	tig00004860	7	19.3		No	73	
**11**	tig00000032	8	39.5	100	No	62	59
**12**	tig00000644	8	83.1		No	62	
**13**	tig00004861	8	44.4		No	62	
**14**	tig00000643	8	103.0		Yes	61	
**15**	tig00000642b	8	24.5		No	61	
**16**	tig00000645	9	105.2	Unknown	Yes	71	60
**17**	tig00000168	9	29.8		No	68	
**18**	tig00000002	10	22.9	Unknown	No	75	61
**19**	tig00000082	10	59.5		Yes	73	
**20**	tig00000152	11	20.9	Unknown	No	71	61
**21**	tig00000133	11	6.1		No	70	
**22**	tig00000115	2 or 7	14.6		No	74	
**23**	tig00000027	unitig_2133 or 8	95.7		No	62	

Supplementary Table 4. Summary of Nanopore assembly of *T. brucei* centromeres.

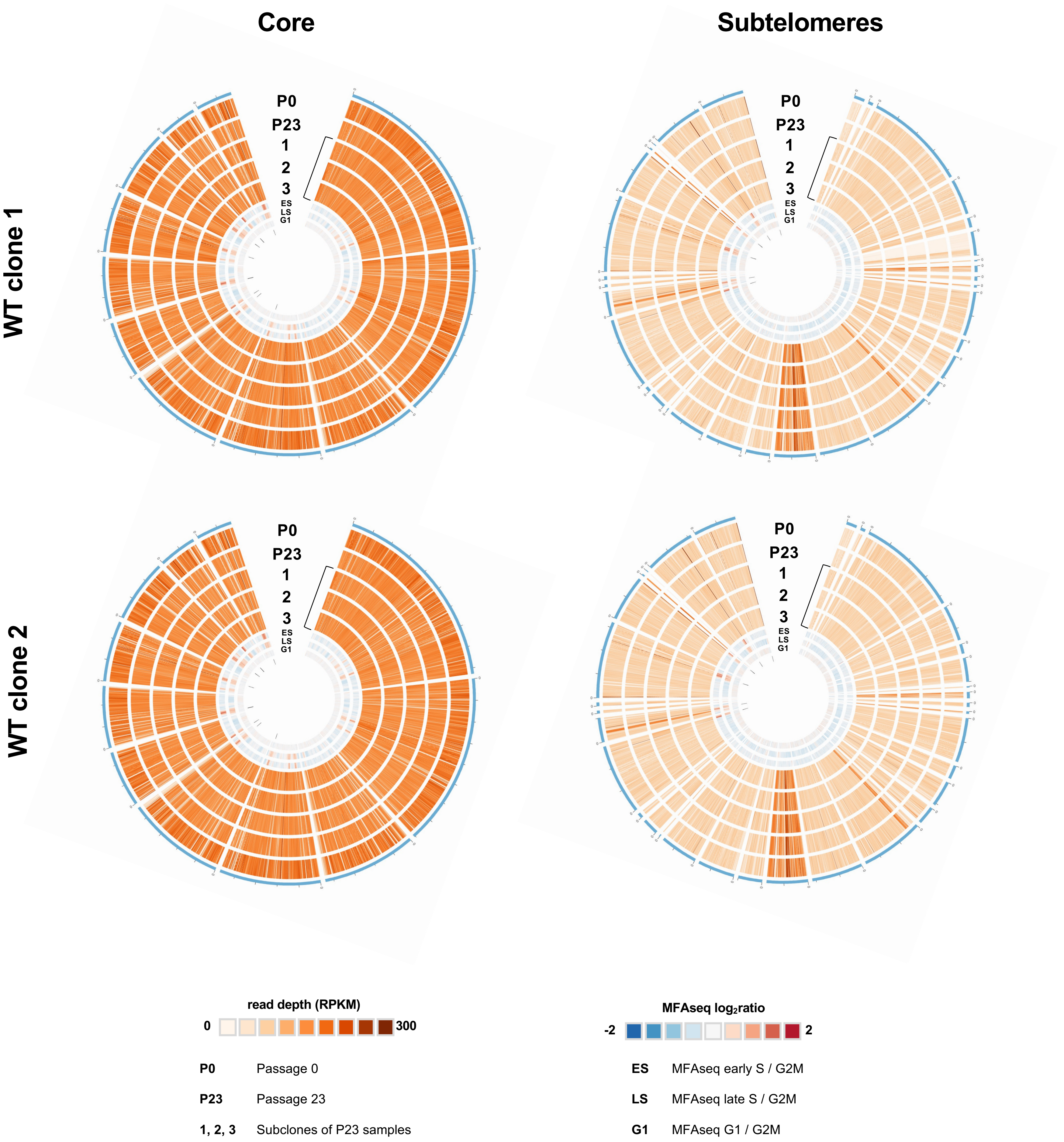
Core
Subtelomeres
WT clone 1
WT clone 2
P0
P23
1
2
3
ES
LS
G1
read depth (RPKM)
0
300
MFAseq log$_2$ratio
-2
2
P0 Passage 0
P23 Passage 23
1, 2, 3 Subclones of P23 samples
ES MFAseq early S / G2M
LS MFAseq late S / G2M
G1 MFAseq G1 / G2M

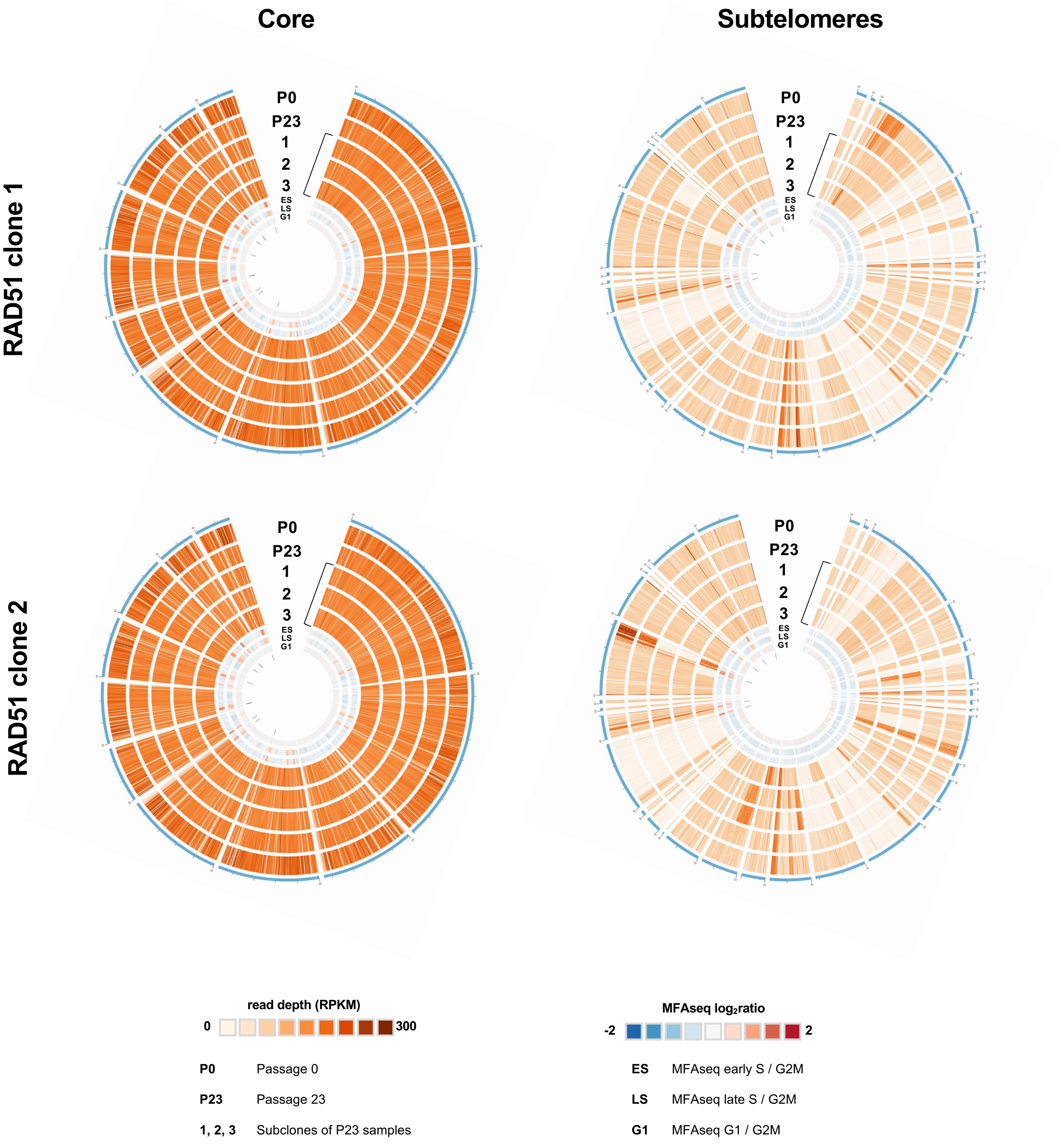
Core
Subtelomeres
RAD51 clone 1
RAD51 clone 2
P0
P23
1
2
3
ES
LS
G1
read depth (RPKM)
0
300
MFAseq log2ratio
-2
2
P0 Passage 0
P23 Passage 23
1, 2, 3 Subclones of P23 samples
ES MFAseq early S / G2M
LS MFAseq late S / G2M
G1 MFAseq G1 / G2M

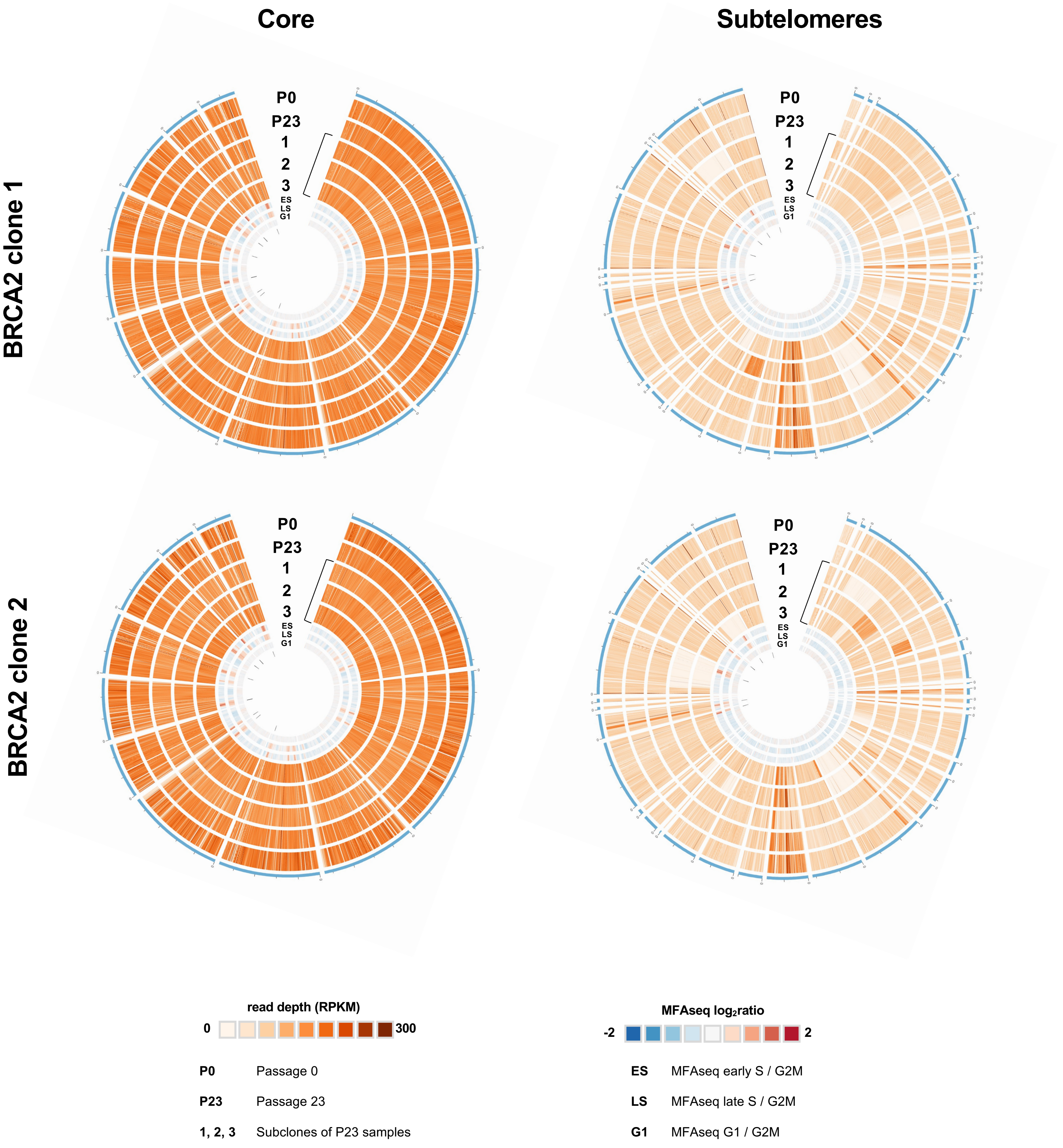

Supplementary Figure 7. Compartmentalisation of stability between the cores and subtelomeres of the *T. brucei* megabase chromosomes. Read depth mapping is shown across the core and subtelomeric compartments in two clones of wild type (WT), RAD51 null mutant and BRCA2 null mutant cells, as follows: as a population before growth (P0), as a population after 23 passages of growth in culture (P23), and in three clones (1, 2, 3) generated from the populations after 23 passages of growth; data is show as a heatmap of RPKM (reads per kb per million reads). The innermost circles show MFA-seq data as plotted in Fig.3.

NO	CONTIG	UP	50BP	BES BODY	70BP	CTR	BES VSG	TTAGGG	ID	CHR	CHR PUB	NOTES ON THE BES OR CONTIG
1	tig00000137	✓	✓	✓		✓	✓	✓	BES10	7 or int	int	no 70bp repeats
2	tig00000644	✓	✓	✓	✓	✓	✓	✓	BES8?	8	8	very few genes in BES body
3	tig00000653		✓	✓	✓	✓	✓	✓	BES1		6	
4	tig00000114		✓	✓	✓	✓	✓		BES12		2	
5	tig00000642	✓	✓	✓	✓	✓	✓		BES15	3	3	two 70bp repeat regions
6	tig00004876	✓	✓	✓	✓	✓			BES11	int	int	177bp repeats
7	tig00004877		✓	✓	✓	✓			BES11		int	
8	tig00004878		✓	✓	✓	✓			BES13		int	
9	tig00000055	✓	✓	✓	✓				BES5	5	5	
10	tig00000156	✓	✓	✓	✓				BES14		7	
11	tig00000157	✓	✓	✓	✓				BES4		int	
12	tig00000658	✓	✓	✓	✓				BES7	int	6	177bp repeats
13	tig00004879	✓	✓	✓	✓				BES13	int	int	177bp repeats and telomere at the 5’ end
14	tig00000116		✓	✓	✓				BES3		4	
15	tig00000652	✓	✓	✓					BES1		6	

Supplementary Table 5. Summary of Nanopore assembly of *T. brucei* VSG expression sites, including upstream 50 bp repeats and subtelomere sequence and inclusion of the telomere tract.

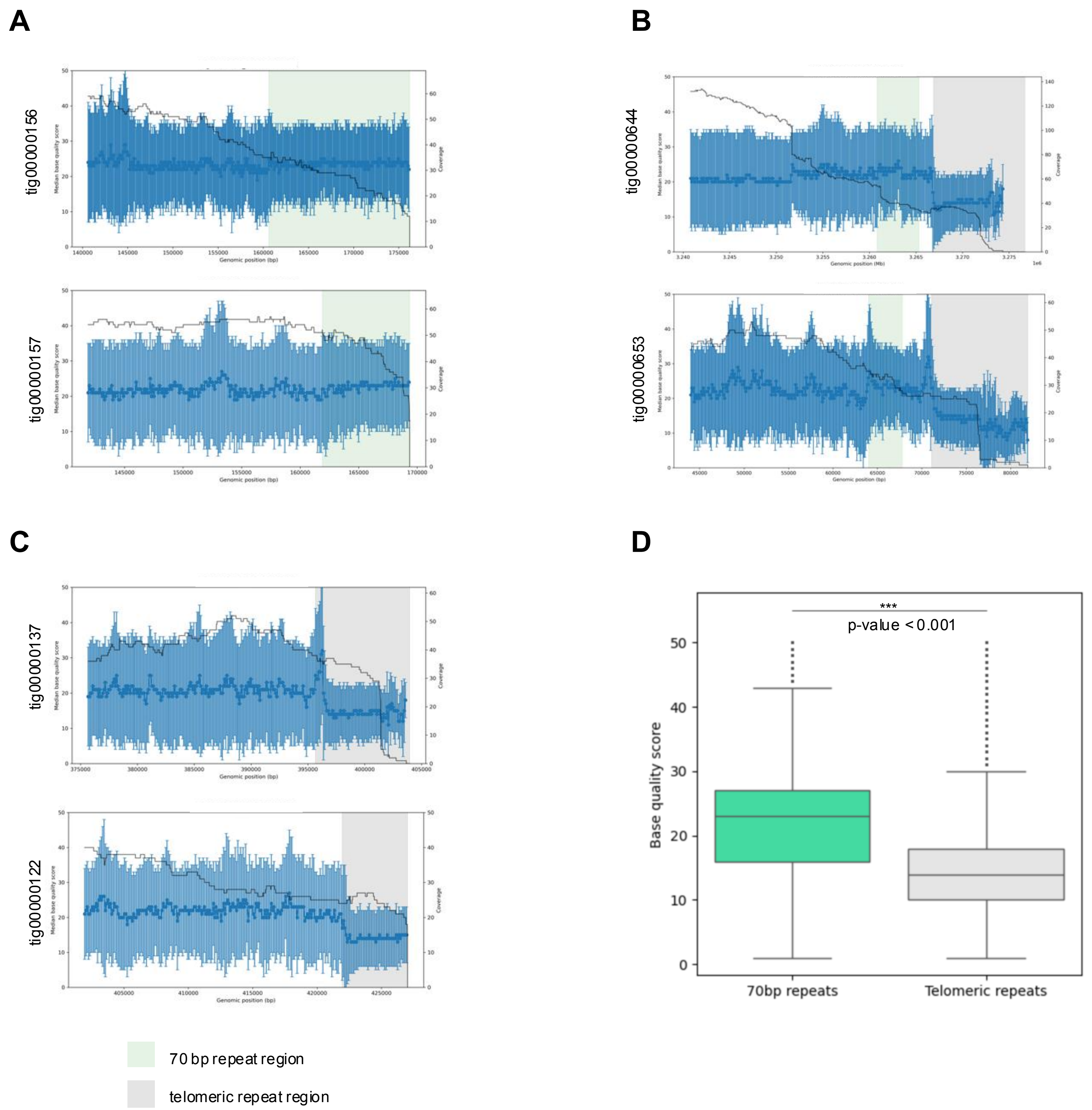

Supplementary Figure 8. Telomeric repeats of *T. brucei* represent a challenge for Nanopore DNA sequencing. Median base quality, along with the interquartile range, is plotted for representative 70 bp- and -telomeric repeat-containing genomic regions; only >10 kb reads represented. **A.** Two examples of truncated 70 bp regions (green) with flanking sequence. **B.** Examples of full-length 70 bp repeat regions (green) as well as telomeric repeats (grey). **C.** Examples of other telomeric repeat-containing genomic regions (grey). **D.** Boxplots showing the distribution of base qualities across all 70 bp regions and all telomeric regions; p-value < 0.001, significance tested using a Mann-Whitney U test (scipy package, v.1.10.1). Source data are provided as source data file.

A

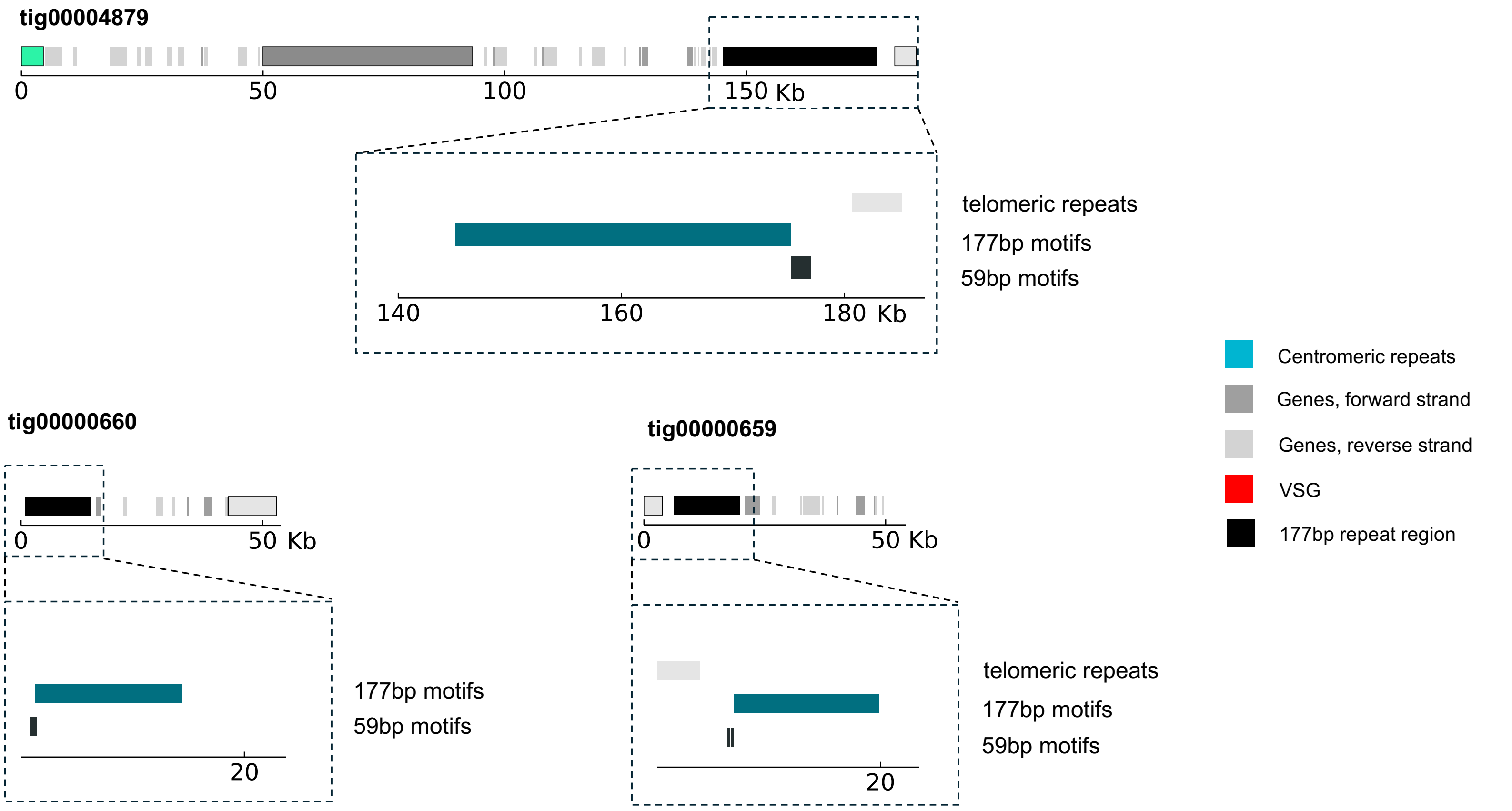

B

TIG	CHR	177BP	59BP
**69**	1	0	20
**654**	1	0	1
**111**	2	2	0
**648**	6	1307	244
**645**	9 (3A)	51	0
**168**	9 (3B)	58	200
**82**	10 (3A)	284	5
**2**	10 (3B)	1	0
**152**	11 (3A)	59	404
**4875**	11 (3A)	3	107
**133**	11 (3B)	5	28

C

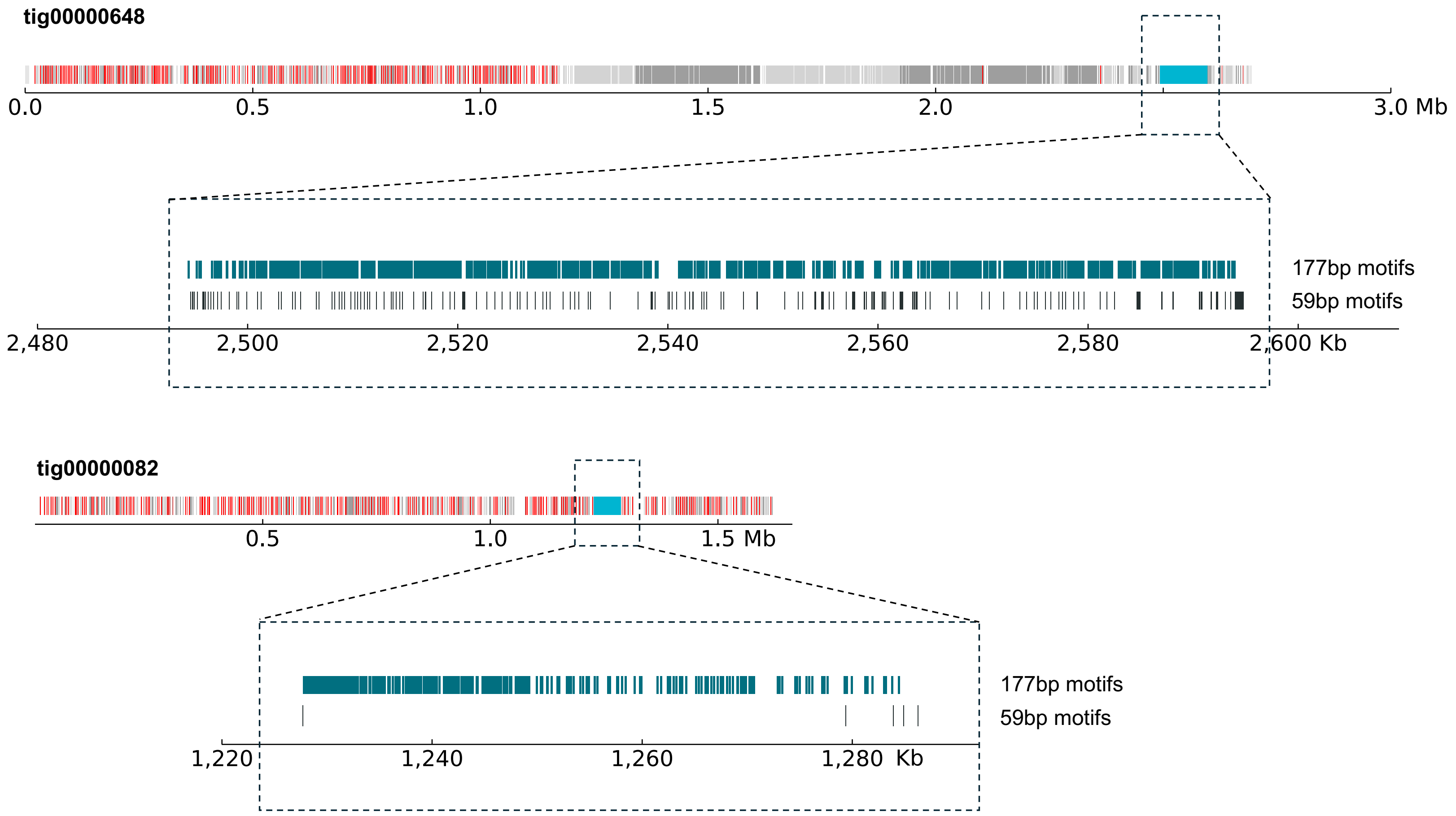

Supplementary Figure 9. 177 bp and 59 bp repeats are found in both the sub-megabase chromosomes and in the centromeres of the megabase chromosomes. A. Organisation of 177 bp and 59 bp repeats in three Nanopore contigs (tigs) of sub-megabase chromosomes; the location of these features is shown relative to the genes within the contigs and a telomere. **B.** Summary of Nanopore contigs containing assembled centromeres in which 177 bp and 59 bp repeats can be detected; the number of the repeat motifs in each centromere is indicated. **C.** Two Nanopore contigs of parts of megabase chromosomes, showing the extent of 177 bp and 59 bp repeats within the putatively fully assembled centromeres; core genes are shown in grey and *VSG*s in red.

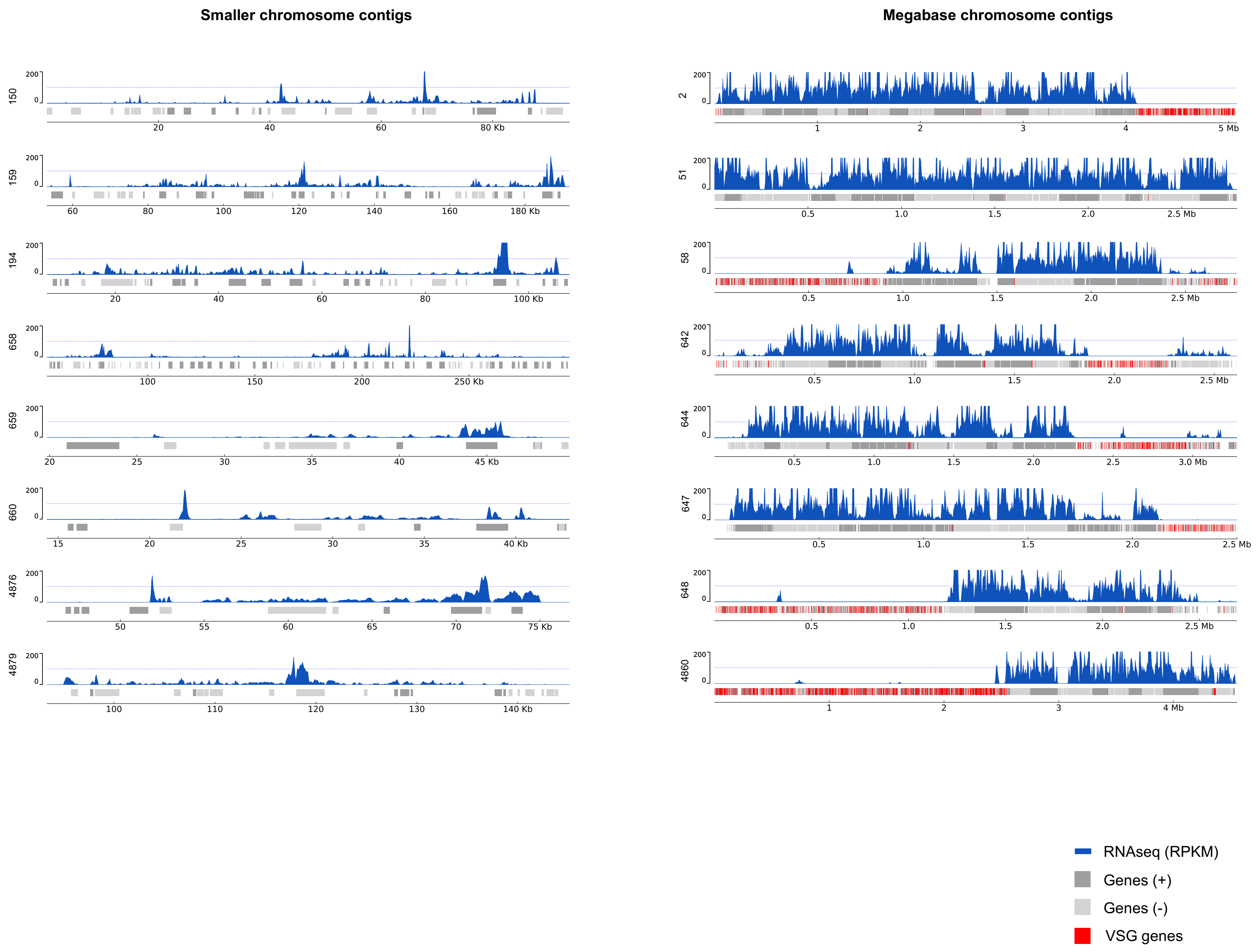

Supplementary Figure 10. A comparison of transcript abundance derived from the sub-megabase chromosomes and from the core and subtelomere compartments of the megabase chromosomes. A comparison of RNA-seq mapping to the non-repetitive components of eight sub-megabase chromosome Nanopore contigs (tigs, smaller chromosomes), excluding the VSG BESs, and to eight megabase chromosome Nanopore contigs that span the core and subtelomeric compartments; in all cases *VSG*s are in red and other genes in dark or light grey depending on transcription direction. RNA-seq mapping is shown as RPKM (reads per kb per million reads mapped), and the blue line denotes 100 RPKM.
