# Supplementary material for: Nanopore sequencing reveals that DNA replication compartmentalisation dictates genome stability and instability in Trypanosoma brucei
Source: Nat Commun. 2025 Jan 16;16:751. doi: 10.1038/s41467-025-56087-3 (PMC11739655; doi:10.1038/s41467-025-56087-3)
Supplement: Supplementary file 2 — Description of Supplementary Data files [file 41467_2025_56087_MOESM2_ESM.docx]

**Description of additional Supplementary Data files**

**File Name:** Supplementary Data 1

**Description:** Description and Analysis of 72 genes and pseudogenes predicted in the Nanopore assembly that were not predicted in the Muller genome.
