# Supplementary material for: Nanopore sequencing reveals that DNA replication compartmentalisation dictates genome stability and instability in Trypanosoma brucei
Source: Nat Commun. 2025 Jan 16;16:751. doi: 10.1038/s41467-025-56087-3 (PMC11739655; doi:10.1038/s41467-025-56087-3)
Supplement: Supplementary file 4 — Reporting Summary [file 41467_2025_56087_MOESM4_ESM.pdf]

nature portfolio

Corresponding author(s): Richard McCulloch

Last updated by author(s): May 29, 2024

Reporting Summary

Nature Portfolio wishes to improve the reproducibility of the work that we publish. This form provides structure for consistency and transparency in reporting. For further information on Nature Portfolio policies, see our Editorial Policies and the Editorial Policy Checklist.

Statistics

For all statistical analyses, confirm that the following items are present in the figure legend, table legend, main text, or Methods section.

n/a	Confirmed	
☐	☒	The exact sample size (*n*) for each experimental group/condition, given as a discrete number and unit of measurement
☐	☒	A statement on whether measurements were taken from distinct samples or whether the same sample was measured repeatedly
☐	☒	The statistical test(s) used AND whether they are one- or two-sided<br>*Only common tests should be described solely by name; describe more complex techniques in the Methods section.*
☒	☐	A description of all covariates tested
☒	☐	A description of any assumptions or corrections, such as tests of normality and adjustment for multiple comparisons
☐	☒	A full description of the statistical parameters including central tendency (e.g. means) or other basic estimates (e.g. regression coefficient) AND variation (e.g. standard deviation) or associated estimates of uncertainty (e.g. confidence intervals)
☒	☐	For null hypothesis testing, the test statistic (e.g. *F*, *t*, *r*) with confidence intervals, effect sizes, degrees of freedom and *P* value noted<br>*Give P values as exact values whenever suitable.*
☒	☐	For Bayesian analysis, information on the choice of priors and Markov chain Monte Carlo settings
☒	☐	For hierarchical and complex designs, identification of the appropriate level for tests and full reporting of outcomes
☒	☐	Estimates of effect sizes (e.g. Cohen's *d*, Pearson's *r*), indicating how they were calculated

Our web collection on statistics for biologists contains articles on many of the points above.

Software and code

Policy information about availability of computer code

Data collection	None
Data analysis	NanoPlot (1.30.0), QUAST (5.0.2), minimap2 (2.22), blast (2.12), canu (1.8), pilon (1.23), bwa (0.7.17), trf (4.09), FIMO (5.0.5), guppy (3.3.3. Linux CPU), BUSCO (3.5.2), OrthoMCL (Galaxy version 1.0.0), samtools (1.19), circos (0.69-8), pygenometracks (3.8), bedtools (2.30.0), deeptools (3.5.1), HISAT2 (2.2.1), trim galore (0.6.10), R (4.3.3), karyoploteR (1.28.0), rtracklayer (1.62.0), dplyr (1.1.4), ggplot2 (3.5.0), GridExtra (2.3), Globus genomics via TriTrypDB (Feb 2021 - FastQC, Trimmomatic, Bowtie2, Deeptools and Bedtools)

For manuscripts utilizing custom algorithms or software that are central to the research but not yet described in published literature, software must be made available to editors and reviewers. We strongly encourage code deposition in a community repository (e.g. GitHub). See the Nature Portfolio guidelines for submitting code & software for further information.

Data

Policy information about availability of data

All manuscripts must include a data availability statement. This statement should provide the following information, where applicable:

- Accession codes, unique identifiers, or web links for publicly available datasets
- A description of any restrictions on data availability
- For clinical datasets or third party data, please ensure that the statement adheres to our policy

Nanopore and Illumina reads have been deposited to the NCBI Sequence Read Archive (SRA) under project number PRJNA962304. The assembled genome is available at the EMBO-EBI European nucleotide archive, accession number PRJEB75536. Further data are available under accession number PRJNA962304.

Research involving human participants, their data, or biological material

Policy information about studies with human participants or human data. See also policy information about sex, gender (identity/presentation), and sexual orientation and race, ethnicity and racism.

Reporting on sex and gender	N/A
Reporting on race, ethnicity, or other socially relevant groupings	N/A
Population characteristics	N/A
Recruitment	N/A
Ethics oversight	N/A

Note that full information on the approval of the study protocol must also be provided in the manuscript.

Field-specific reporting

Please select the one below that is the best fit for your research. If you are not sure, read the appropriate sections before making your selection.

☒ Life sciences ☐ Behavioural & social sciences ☐ Ecological, evolutionary & environmental sciences

For a reference copy of the document with all sections, see nature.com/documents/nr-reporting-summary-flat.pdf

Life sciences study design

All studies must disclose on these points even when the disclosure is negative.

Sample size	N/A
Data exclusions	N/A
Replication	N/A
Randomization	N/A
Blinding	N/A

Reporting for specific materials, systems and methods

We require information from authors about some types of materials, experimental systems and methods used in many studies. Here, indicate whether each material, system or method listed is relevant to your study. If you are not sure if a list item applies to your research, read the appropriate section before selecting a response.

Materials & experimental systems

n/a	Involved in the study
☒	☐ Antibodies
☒	☐ Eukaryotic cell lines
☒	☐ Palaeontology and archaeology
☒	☐ Animals and other organisms
☒	☐ Clinical data
☒	☐ Dual use research of concern
☒	☐ Plants

Methods

n/a	Involved in the study
☐	☒ ChIP-seq
☒	☐ Flow cytometry
☒	☐ MRI-based neuroimaging

Plants

Seed stocks: N/A

Novel plant genotypes: N/A

Authentication: N/A

ChIP-seq

Data deposition

☒ Confirm that both raw and final processed data have been deposited in a public database such as GEO.

☒ Confirm that you have deposited or provided access to graph files (e.g. BED files) for the called peaks.

Data access links
May remain private before publication.
DRIP-seq (a form of ChIP-seq) sequences are available under accession number PRJEB21868 and previously published: Briggs, E., Crouch, K., Lemgruber, L., Lapsley, C. & McCulloch, R. Ribonuclease H1-targeted R- loops in surface antigen gene expression sites can direct trypanosome immune evasion. PLoS genetics 14, e1007729 (2018)

Files in database submission
Provide a list of all files available in the database submission.

Genome browser session (e.g. UCSC)
N/A

Methodology

Replicates
Described here: DRIP-seq (a form of ChIP-seq) sequences are available under accession number PRJEB21868 and previously published: Briggs, E., Crouch, K., Lemgruber, L., Lapsley, C. & McCulloch, R. Ribonuclease H1-targeted R- loops in surface antigen gene expression sites can direct trypanosome immune evasion. PLoS genetics 14, e1007729 (2018)

Sequencing depth
Described here: DRIP-seq (a form of ChIP-seq) sequences are available under accession number PRJEB21868 and previously published: Briggs, E., Crouch, K., Lemgruber, L., Lapsley, C. & McCulloch, R. Ribonuclease H1-targeted R- loops in surface antigen gene expression sites can direct trypanosome immune evasion. PLoS genetics 14, e1007729 (2018)

Antibodies
Described here: DRIP-seq (a form of ChIP-seq) sequences are available under accession number PRJEB21868 and previously published: Briggs, E., Crouch, K., Lemgruber, L., Lapsley, C. & McCulloch, R. Ribonuclease H1-targeted R- loops in surface antigen gene expression sites can direct trypanosome immune evasion. PLoS genetics 14, e1007729 (2018)

Peak calling parameters
Described here: DRIP-seq (a form of ChIP-seq) sequences are available under accession number PRJEB21868 and previously published: Briggs, E., Crouch, K., Lemgruber, L., Lapsley, C. & McCulloch, R. Ribonuclease H1-targeted R- loops in surface antigen gene expression sites can direct trypanosome immune evasion. PLoS genetics 14, e1007729 (2018)

Data quality
Described here: DRIP-seq (a form of ChIP-seq) sequences are available under accession number PRJEB21868 and previously published: Briggs, E., Crouch, K., Lemgruber, L., Lapsley, C. & McCulloch, R. Ribonuclease H1-targeted R- loops in surface antigen gene expression sites can direct trypanosome immune evasion. PLoS genetics 14, e1007729 (2018)

Software
Described here: DRIP-seq (a form of ChIP-seq) sequences are available under accession number PRJEB21868 and previously published: Briggs, E., Crouch, K., Lemgruber, L., Lapsley, C. & McCulloch, R. Ribonuclease H1-targeted R- loops in surface antigen gene expression sites can direct trypanosome immune evasion. PLoS genetics 14, e1007729 (2018)
